# Prognostic Role of 14F7 Mab Immunoreactivity against N-Glycolyl GM3 Ganglioside in Colon Cancer

**DOI:** 10.1155/2014/482301

**Published:** 2014-01-30

**Authors:** Tania Lahera, Adanays Calvo, Griselda Torres, Charles E. Rengifo, Santiago Quintero, María del Carmen Arango, Debora Danta, José M. Vázquez, Xiomara Escobar, Adriana Carr

**Affiliations:** ^1^Department of Basic Research and Immunology, National Institute of Oncology and Radiobiology, 29 and F Street Vedado, 10400 Havana, Cuba; ^2^Department of Cell Biology and Tissues Banking, National Institute of Oncology and Radiobiology, 29 and F Street Vedado, 10400 Havana, Cuba; ^3^Department of Pathology, Manuel Fajardo Hospital, Zapata and D Street Vedado, 10400 Havana, Cuba; ^4^Department of Pathology, National Institute of Oncology and Radiobiology, 29 and F Street Vedado, 10400 Havana, Cuba; ^5^Department of Surgery, National Institute of Oncology and Radiobiology, 29 and F Street Vedado, 10400 Havana, Cuba; ^6^Research and Development Direction, Center of Molecular Immunology, 216 Street and 15 Avenue Atabey, Playa, P.O. Box 16040, 11600 Havana, Cuba

## Abstract

*Purpose*. To assess the prognostic role of 14F7 Mab immunoreactivity, against N-Glycolyl GM3 ganglioside, in patients with colon cancer (CC) and to evaluate the relationship between its expression and clinicopathological features. *Methods*. Paraffin-embedded specimens were retrospectively collected from 50 patients with CC operated between 2004 and 2008. 14F7 Mab staining was determined by immunohistochemistry technique and its relation with survival and clinicopathologic features was evaluated. *Results*. The reactivity of 14F7 Mab was detected in all cases. Most cases had high level of immunostaining (70%) that showed statistical correlation with TNM stage (*P* = 0.025). In univariate survival analysis, level of 14F7 Mab immunoreactivity (*P* = 0.0078), TNM Stage (*P* = 0.0007) and lymphovascular invasion (0.027) were significant prognostic factors for overall survival. Among these variables, level of 14F7 Mab immunoreactivity (HR = 0.268; 95% CI  0.078–0.920; *P* = 0.036) and TNM stage (HR = 0.249; 95% CI 0.066–0.932; *P* = 0.039) were independent prognostic factors on multivariate analysis. *Conclusions*. This study is the first approach on the prognostic significance of 14F7 Mab immunoreactivity in patients with colon adenocarcinoma and this assessment might be used in the prognostic estimate of CC, although further studies will be required to validate these findings.

## 1. Introduction

Colon cancer (CC) is the third most common cancer in both genders worldwide and the second cause of cancer-related death in western countries, only overcome by lung cancer [1, 2]. In Cuba, CC is one of the most frequent tumors and the third cause of death by cancer in both men and women [3].

Despite the significant advances in the treatment and knowledge of the colon tumor biology over the last decade, the 5-year survival after surgery varies from 90% to 10%, depending on stage and progression of disease [2, 4]. Therefore, it is important to increase our understanding of the molecular changes leading to development, spread, and metastasis and to identify potential prognostic and predictive markers for the disease.

To date, TNM (tumor-node metastasis) staging, based on the American Joint Committee on Cancer (AJCC) and the International Union against Cancer (UICC) classifications, has been the most important prognostic marker in CC [5]. However, this classification provides limited information, since cancer outcomes vary significantly among patients within the same stage. Therefore, numerous biomarkers have been investigated over the past years [6]. In this regard, ganglioside is one of the molecules under evaluation as biomarker and target for therapy [7].

Gangliosides are sialic acid-containing glycosphingolipids expressed in the plasmatic membrane of vertebrate's cell. N-Acetylneuraminic acid (NeuAc) is the most common in humans, while N-glycolylneuraminic acid (NeuGc) is not usually detected in normal tissues due to a specific inactivating mutation in the cytidine monophospho-N-acetylneuraminic acid hydroxylase (CMP-NeuAc hydroxylase) gene [8, 9]. However, the expression of N-glycolyl-containing gangliosides has been found in a variety of human malignancies [10, 11], suggesting its possible role in oncogenic process and becoming in attractive targets for cancer immunotherapy.

The expressions of NeuGcGM3 ganglioside have been previously reported in different tumors using 14F7 Mab [12–15], a murine IgG1 highly specific against the N-Glycolyl-GM3 ganglioside produced by the Center of Molecular Immunology, Havana, Cuba [10]. Blanco et al. evaluated the 14F7 Mab immunostaining in digestive system tumors, including some specimens of colon adenocarcinoma [16].

The objective of this study was to assess the prognostic role of 14F7 Mab immunoreactivity in patients with CC and evaluate the relationship between its expression and clinicopathological features.

## 2. Materials and Methods

### 2.1. Patients and Samples

We studied tumor specimens from 50 patients diagnosed with colon cancer who underwent tumor surgical resection at the National Institute of Oncology, Havana, Cuba, between 2004 and 2008. Tissues samples were processed following standard histological techniques described elsewhere [17].

All routinely stained sections were reviewed by two pathologists, who did all histopathologic classifications including stage, grade of differentiation, tumor type (mucinous or nonmucinous), peritumoral inflammation, degree of cell pleomorphism, and mitotic index; these last two were previously described [14].

Clinical data were obtained by reviewing the patient records. This research was approved by the ethical committee of the institute.

### 2.2. Immunohistochemistry

Briefly, 4 *μ*m thick sections were cut from formalin-fixed and paraffin-embedded blocks of surgical specimens. Subsequently the sections were dewaxed in xylene and rehydrated through a graded series of ethanol. Endogenous peroxidase activity was inhibited by incubating the sections in 0.3% hydrogen peroxide in methanol for 10 minutes followed by Tris-buffered saline wash. Nonspecific labeling was blocked with bovine serum albumin for 30 min. As primary antibody, we used the 14F7Mab, a murine IgG1 highly specific against N-Glycolyl-GM3 ganglioside. This antibody was produced by the Center of Molecular Immunology, Havana, Cuba [10]. Sections were incubated with 14F7 Mab (12 *μ*g/mL) for 30 minutes at room temperature. Then a kit Universal Dako LSAB+System-HRP (code K0679, Dako, Denmark) was applied to all sections according to the manufacturer's recommendations. Finally, the sections were counterstained with Mayer's hematoxylin, dehydrated through ascending ethanols to xylene, and mounted with Eukitt (Kinder GmbH & Co., Freiburg, Germany). As a negative control, the primary antibody was omitted and replaced with Tris-buffered saline. Breast carcinoma tissue was used as a positive control [10]. Both tumoral and normal tissue area surrounding were evaluated.

### 2.3. Evaluation of Immunostaining

Immunohistochemical results were evaluated according to proportion of stained cells and intensity of 14F7 Mab reactivity. The proportion of stained cells was graded on a scale of 0–3 (0, no staining; 1, 1–50%; 2, 51–75%; and 3, 76–100%). The staining intensity was graded on a scale of 0–3; 0, no staining; 1, weak staining; 2, moderate staining; and 3, strong staining. Subsequently, an immunoreactive scoring (IRS) was obtained by multiplying the two previously mentioned parameters. The cutoff level of 14F7 immunostaining was defined as a dichotomous variable of low level (IRS < 6) or high level (IRS ≥ 6). All slides were assessed by two trained observers who did not have knowledge of clinical characteristics or outcomes.

### 2.4. Statistical Analysis

The relationships between 14F7 Mab immunoreactivity and clinicopathologic variables were analyzed using the chi-square test. Correlations were determined using Spearman's test.

Survival rates were estimated by the Kaplan-Meier method and compared with the log-rank test. For multivariate analyses, the Cox regression model was used to identify independent prognostic factors for overall survival (OS) and disease-free survival (DFS). The model included all variables to have significant prognostic value in univariate analysis (log-rank test).

OS was measured from the date of surgery to death for any cause or last follow-up and was calculated for all patients. DFS was measured from the date of surgery to the date of second cancer, locoregional recurrence, distant metastases, and death for any cause or last follow-up and was calculated in patients with disease stages I–III. All data on survival and disease-free survival were updated in July 2013. The median follow-up time was 62 months.

A *P* value *< *0.05 was considered statistically significant. Statistical analysis was carried out using SPSS (version 11.5; SPSS Inc., Chicago, IL).

## 3. Results

### 3.1. Patient Characteristics

Clinical and pathological characteristics of patients are summarized in Table 1. The study population had a median age of 63 years (range: 36–85 years). According to histopathological type, all tumors were adenocarcinomas and the majority was of a moderate histological grade (62%). Perineural invasion was detected in one patient.

### 3.2. 14F7 Mab Immunostaining

**Figure 1:**
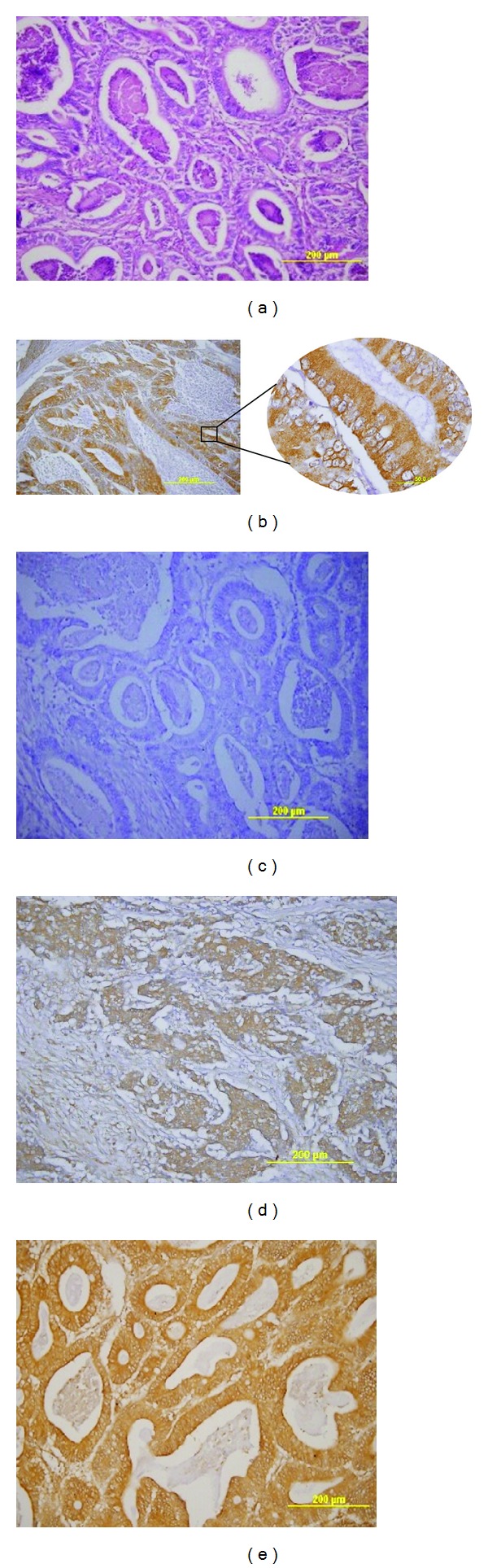
14F7 Mab immunostaining in colon adenocarcinoma sections. (a) Hematoxylin and eosin staining. (b) 14F7 Mab positive reactivity in membrane and cytoplasm of tumor cells with finely granular pattern. (c) Negative control. (d) Low level of 14F7 Mab immunostaining. (e) High level of 14F7 Mab immunostaining. Original magnification 200x (inset 1000x magnification).

Representative images of 14F7 Mab immunoreactivity are illustrated in Figure 1. The immunostaining was observed in all cases, with variable intensity and proportion of stained cells. Most specimens had strong intensity (68%) and more than 75% of positive cells (62%) as shown in Table 2. A moderate correlation was found between percentage of positive tumor cells and staining intensity (Spearman's correlation coefficient 0,606; *P* = 0,000). According to IRS, most cases showed high level of immunostaining (70%). The reactivity was observed on both membrane and cytoplasm of tumor cells with a staining pattern finely granular. In a few cases (six specimens) a moderate immunoreactivity in normal tissue areas was detected.

### 3.3. Relation of 14F7 Mab Immunostaining with Clinicopathologic Variables

The relation of 14F7 Mab immunostaining with clinicopathologic variables is shown in Table 3. No significant differences were observed with age, sex, tumor location, grade of differentiation, mucinous type, mitotic index, pleomorphism grade, peritumoral inflammation, or lymphovascular invasion. However, the level of immunoreactivity showed statistical correlation with TNM stage (*P* = 0,025 and Spearman *r* = 0,317). When cases were analyzed independently, according to intensity or extent of staining (data not shown), no significant associations with clinicopathologic features were noted, except for the positive relation between proportion of stained cells and TNM stage (*P* = 0,038).

### 3.4. Survival Analysis

In survival analysis, there was a significant difference in the 5-year OS rates between high and low 14F7 Mab immunostaining (40% versus 86,7%; *P* = 0,002). Furthermore, patients with high level of 14F7 Mab immunoreactivity had significantly impaired 5-year DFS (*P* = 0,046) than those with low level (60,9% versus 92,3%).

**Figure 2:**
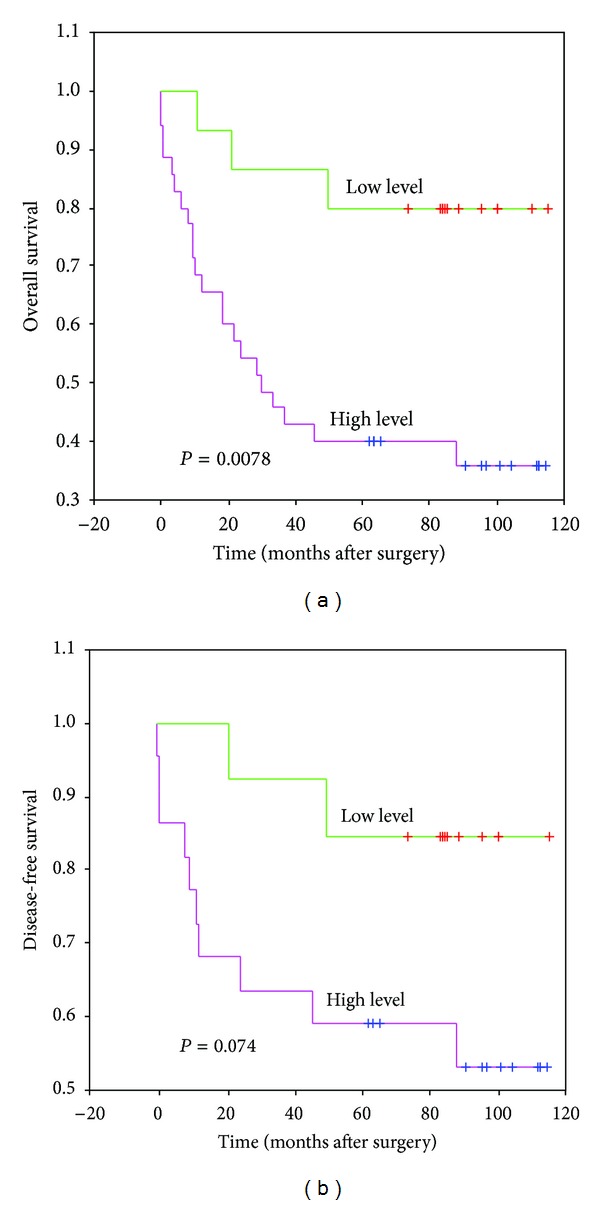
Kaplan-Meier curves for overall survival and disease-free survival according to 14F7 Mab immunostaining level. Statistical analysis by log-rank test.

Kaplan-Meier curves are represented in Figure 2. Immunostaining was associated significantly with OS (*P* = 0,0078) while no significant relation was demonstrated with DFS, although a trend existed (*P* = 0,0745).

The results of univariate and multivariate survival analysis are summarized in Table 4. Univariate analysis showed that level of 14F7 Mab immunostaining (*P* = 0,0078), TNM stage (*P* = 0,0007), and lymphovascular invasion (0,027) were significant prognostic factors for OS. Among these variables, level of 14F7 Mab immunostaining (HR = 0,268; 95% CI 0,078–0,920; *P* = 0,036) and TNM stage (HR = 0,249; 95% CI 0,066–0,932; *P* = 0,039) were independent prognostic factors on multivariate analysis. For DFS, tumor location was significant prognostic factor (*P* = 0,036) since patients with sigmoides tumor had poor survival. However, a trend existed for the level of 14F7 Mab immunostaining (*P* = 0,074). As only one variable was significant in univariate analysis, multivariate analysis was not performed.

## 4. Discussion

Given the limited impact of conventional factors in CC, it is necessary to identify new prognostic biomarkers that provide information concerning the natural history of this disease. The present study is the first to evaluate the prognostic significance of 14F7 Mab immunostainingin patients with colon adenocarcinoma.

The 14F7 Mab immunoreactivity, against NeuGcGM3, has been previously reported in some tumors including breast carcinoma [11], skin neoplasms [12], lung cancer [14], and neuroectodermal tumors [15].

In our research, we used formalin-fixed and paraffin-embedded tissues, which is common in retrospective and long-term survival studies. However, as the routine tissues processing could damage the structure of gangliosides, further studies in frozen samples are recommended to confirm these results.

Although the presence of NeuGcGM3 in tumors has been demonstrated, the mechanisms that support its expression have been controversial. Some studies suggest that its presence in human cancer is due to metabolic incorporation of dietary NeuGc, related with changes in the metabolism of tumor cells. It is well described that cells can process exogenous sialic acids from the extracellular environment and use them for their own glycoconjugates [18, 19].

Furthermore, our data showed a moderate 14F7 Mab reaction in some normal glands surrounding the tumor. This is in line with previous studies that reported a limited recognition of 14F7 Mab in normal tissues [12–14, 16]. A possible mechanism for this finding is that normal eukaryotic cells were able to take in a portion of ingested NeuGc and process it for their own glycoconjugates [18, 20], although other researches are needed in this regard.

In this study, high 14F7 Mab immunostaining was significantly associated with advanced TNM stage (*P* = 0,025), which is characterized by the presence of cancer cells in regional lymph nodes and evidence for metastases. Although our findings suggest the role of NeuGcGM3 ganglioside in the oncogenic process, tumor growth and progression, further studies in larger sample with better distribution by each stage are required.

Our data is consistent with a previous report of Scursoni et al., who found a correlation between the expression of NeuGc-GM3, using 14F7 Mabs and more aggressive disease, in neuroectodermal tumors [15].

Previous studies have shown the relevance of this NeuGcGM3 in cancer progression and its capability of modulating CD4 expression on T cells [21–23]. Some properties of this ganglioside have been described including significant inhibition of human dendritic cells differentiation by inducing apoptosis in precursor cells [24], modification of CD4 expression [25], and promotion of Th2 differentiation pattern in T lymphocytes [26]. In fact, this molecule has been described as one of the most immunosuppressive gangliosides [27].

Other reports have studied a link between NeuGc expression and tumor progression [28]. Bardor et al. [18] and Nguyen et al. [29] showed that uptake of Neu5Gc into human tumor cells in vitro and delivery into the cytosolic compartment via a lysosomal transporter are enhanced by high cell growth rates. Furthermore, hypoxic conditions in tumors can upregulate expression of specific lysosomal transporter [30]. The tumor progression by stimulating inflammation via binding to Neu5Gc-positive tumor cells is another mechanism described. Hedlund et al. [31] found that the combination of tumor-associated Neu5Gc and circulating anti-Neu5Gc antibodies promotes tumor growth, by inducing weak inflammation, causing infiltration of inflammatory cells and enhanced angiogenesis.

In survival analysis, we reported that higher 14F7 Mab immunostaining was significantly associated with impaired OS (in univariate and multivariate analysis) and DFS (in univariate analysis), suggesting its potential use in the prognostic estimate of colon adenocarcinoma. No similar studies have been previously described in this regard.

Only few reports in lung cancer have evaluated the prognostic role of this molecule. Blanco et al. [14] and van Cruijsen et al. [28] found that NeuGc-GM3 expression was associated with better survival in non-small-cell lung cancer (NSCLC), while Hayashi et al. [32] shown an opposite data. Several factors can account for the controversial results. One possibility is the differences in predominant histological type, as in the first two cases was it squamous subtype, while in the third it was adenocarcinoma, resembling our study. Other factors like the number of patients evaluated and the distribution according to clinical stage as well as the specificity of the antibodies might lead to differences in results.

In current research, univariate survival analysis confirmed the poor OS associated with TNM stage and lymphovascular invasion, two putative pathologic markers of invasion and adverse outcome in CC [33].

Gangliosides have been considered attractive targets for cancer immunotherapy based on their higher abundance in tumors when compared with the corresponding normal tissue. In Cuba, two NeuGc ganglioside-based vaccines are currently tested. Several clinical trials have been performed with the anti-NeuGc-containing gangliosides anti-idiotype monoclonal antibody racotumomab (also known as 1E10) and the conjugated NeuGcGM3/VSSP (very small size proteoliposomes) nanoparticle vaccine for immunotherapy of melanoma, breast, and lung cancer. Both vaccines targeted to NeuGc-GM3 ganglioside have acceptable safety outcomes and are able to induce specific humoral and cellular immune responses. Preliminary evidence suggested that these vaccines may have a positive influence on survival in patients with immune response to NeuGcGM3 antigen [7, 34–36].

## 5. Conclusion

This study is the first approach on the prognostic significance of 14F7 Mab immunoreactivity, against N-Glycolyl GM3 ganglioside, in patients with colon adenocarcinoma. The immunostaining correlated significantly with TNM stage and it was an independent prognostic indicator of overall survival in multivariate survival analysis. The current research suggests that assessment of 14F7 reactivity might be used in the prognostic estimate of colon adenocarcinoma, although our results need to be validated in a larger sample and prospective studies. Furthermore, the role of NeuGcGM3 in tumor biology and its differential expression in tumor cells suggest its potential use as target for immunotherapy.

## Figures and Tables

**Table 1:** Clinicopathologic features of studied patients with colon cancer.

Clinicopathologic features	Number (%)
Age, years (*n* = 47)	
≤60	20 (42,6)
>60	27 (57,4)
Sex (*n* = 50)	
Women	27 (54)
Men	23 (46)
Tumor location (*n* = 49)	
Sigmoid colon	21 (42,9)
Left side colon	3 (6,1)
Transverse colon	6 (12,2)
Right side colon	19 (38,8)
TNM stage (*n* = 50)	
I	11 (22)
II	17 (34)
III	7 (14)
IV	15 (30)
Grade of differentiation (*n* = 50)	
Well	12 (24)
Moderate	31 (62)
Poor	7 (14)
Mucinous type (*n* = 50)	
Yes	13 (26)
No	37 (74)
Mitotic index (*n* = 49)	
Low	36 (73,5)
Moderate	4 (8,2)
High	9 (18,4)
Grade of cell pleomorphism (*n* = 49)	
Low	21 (42,9)
Moderate	27 (55,1)
High	1 (2)
Peritumoral inflammation (*n* = 50)	
Low	23 (46)
High	27 (54)
Lymphovascular invasion (*n* = 50)	
Yes	5 (10)
No	45 (90)

TNM: tumor node metastases.

**Table 2:** 14F7 Mab immunostaining in colon cancer.

14F7 Mab immunostaining (*n* = 50)	Number (%)
Staining intensity	
Weak	3 (6)
Moderate	13 (26)
Strong	34 (68)
Staining extent (%)	
−50%	5 (10)
51–75%	14 (28)
+75%	31 (62)
Level of immunostaining	
Low (IRS < 6)	15 (30)
High (IRS ≥ 6)	35 (70)

IRS: immunoreactive score.

**Table 3:** 14F7 Mab immunostaining in relation to clinicopathologic features in colon cancer.

Clinicopathologic features	Level of immunostaining (IRS)		
	Low	High	P*
Age, years (*n* = 47)			0,098
≤60	8	12	
>60	5	22	
Sex (*n* = 50)			0,596
Women	8	19	
Men	7	16	
Tumor location (*n* = 49)			0,841
Sigmoid colon	5	16	
Left side colon	1	2	
Transverse colon	2	4	
Right side colon	7	12	
TNM stage (*n* = 50)			**0,025**
I-II	12	16	
III-IV	3	19	
Grade of differentiation (*n* = 50)			0,311
Well/moderate	14	29	
Poor	1	6	
Mucinous type (*n* = 50)			0,602
Yes	4	9	
No	11	26	
Mitotic index (*n* = 49)			0,550
Low	10	26	
Moderate-high	4	9	
Grade of cell pleomorphism (*n* = 49)			0,622
Low	6	15	
Moderate-high	8	20	
Peritumoral inflammation (*n* = 50)			0,596
Low	7	16	
High	8	19	
Lymphovascular invasion (*n* = 50)			0,524
Yes	1	4	
No	14	31	

*Chi-square test; IRS: immunoreactive score; TNM: tumor node metastases.

Bold value indicates statistical significance.

**Table 4:** Univariate and multivariate analysis of overall survival and disease-free survival in studied population.

Variable	Overall survival			Disease-free survival
	Univariate	Multivariate		Univariate
	*P *	HR (95% CI)	*P *	*P *
Age	0,177			0,117
Sex	0,551			0,269
Tumor location	0,301			**0,036**
TNM stage	**0,0007**	0,249 (0,066–0,932)	**0,039**	0,879
Grade of differentiation	0,197			0,412
Mucinous type	0,754			0,326
Mitotic index	0,196			0,294
Grade of cell pleomorphism	0,425			0,663
Peritumoral inflammation	0,9122			0,276
Lymphovascular invasion	**0,027**	0,315 (0,087–1,146)	0,080	0,582
Level of 14F7 Mab immunostaining	**0,0078**	0,268 (0,078–0,920)	**0,036**	0,074

HR: hazard ratio; CI: confidence interval; TNM: tumor node metastases.

Bold value indicates statistical significance.
